# SWIFT: Prospective 48-Week Study to Evaluate Efficacy and Safety of Switching to Emtricitabine/Tenofovir From Lamivudine/Abacavir in Virologically Suppressed HIV-1 Infected Patients on a Boosted Protease Inhibitor Containing Antiretroviral Regimen

**DOI:** 10.1093/cid/cis1203

**Published:** 2013-01-29

**Authors:** R. Campo, E. DeJesus, U. F. Bredeek, K. Henry, H. Khanlou, K. Logue, C. Brinson, P. Benson, L. Dau, H. Wang, K. White, J. Flaherty, T. Fralich, B. Guyer, D. Piontkowsky

**Affiliations:** 1 Department of Infectious Diseases, University of Miami School of Medicine, Florida; 2 Department of Infectious Disease, Orlando Immunology Center, Florida; 3 Department of Infectious Diseases, Metropolis Medical, San Francisco, California; 4 Department of Internal Medicine HIV Program, Hennepin County Medical Center, Minneapolis, Minnesota; 5 Medical Institute of Immunology and Infectious Diseases, Los Angeles, California; 6 Department of Medicine, St. Clair Medical Associates, Toronto, Ontario, Canada; 7 Central Texas Clinical Research, Austin, Texas; 8 Be Well Medical Center, Berkley, Michigan; 9 Department of Medical Affairs; 10 Department of Biostatistics; 11 Department of Clinical Virology; 12 Department of Clinical Research, Gilead Sciences, Inc, Foster City, California

**Keywords:** HIV-1, FTC/TDF, 3TC/ABC, virologic failure, switch

## Abstract

***Background.*** In the United States, emtricitabine/tenofovir disoproxil fumarate (FTC/TDF) is a preferred nucleoside reverse transcriptase inhibitor (NRTI) backbone with lamivudine/abacavir (3TC/ABC) as a commonly used alternative. For patients infected with human immunodeficiency virus (HIV-1) virologically suppressed on a boosted protease inhibitor (PI) + 3TC/ABC regimen, the merits of switching to FTC/TDF as the NRTI backbone are unknown.

***Methods.*** SWIFT was a prospective, randomized, open-label 48-week study to evaluate efficacy and safety of switching to FTC/TDF. Subjects receiving 3TC/ABC + PI + ritonavir (RTV) with HIV-1 RNA < 200 c/mL ≥3 months were randomized to continue 3TC/ABC or switch to FTC/TDF. The primary endpoint was time to loss of virologic response (TLOVR) with noninferiority measured by delta of 12%. Virologic failure (VF) was defined as confirmed rebound or the last HIV-1 RNA measurement on study drug ≥200 c/mL.

***Results.*** In total, 311 subjects were treated in this study (155 to PI + RTV + FTC/TDF, 156 to PI + RTV + 3TC/ABC). Baseline characteristics were similar between the arms: 85% male, 28% black, median age, 46 years; and median CD4 532 cells/mm^3^. By TLOVR through week 48, switching to FTC/TDF was noninferior compared to continued 3TC/ABC (86.4% vs 83.3%, treatment difference 3.0% (95% confidence interval, −5.1% to 11.2%). Fewer subjects on FTC/TDF experienced VF (3 vs 11; *P* = .034). FTC/TDF showed greater declines in fasting low-density lipoproteins (LDL), total cholesterol (TC), and triglycerides (TG) with significant declines in LDL and TC beginning at week 12 with no TC/HDL ratio change. Switching to FTC/TDF showed improved NCEP thresholds for TC and TG and improved 10-year Framingham TC calculated scores. Decreased epidermal growth factor receptor (eGFR) was observed in both arms with a larger decrease in the FTC/TDF arm.

***Conclusions.*** Switching to FTC/TDF from 3TC/ABC maintained virologic suppression, had fewer VFs, improved lipid parameters and Framingham scores but decreased eGFR.

***ClinicalTrials.gov identifier.*** NCT00724711.

## Abstract (precis)

Virologic suppression was well maintained when HIV patients receiving 3TC/ABC with a boosted protease inhibitor were switched to emtricitabine/tenofovir disoproxil fumarate (FTC/TDF). Subjects randomized to FTC/TDF) had fewer virologic failures; in addition, improvements in lipids and Framingham risk scores were noted, while slight declines in estimated GFR were observed.

Fixed-dose combination (FDC) antiretrovirals such as emtricitabine/tenofovir disoproxil fumarate (FTC/TDF) and lamivudine/abacavir (3TC/ABC) allow simplification of regimens to potentially improve outcomes by augmenting adherence [1]. Comparative studies of FTC/TDF to 3TC/ABC-containing regimens tend to favor the FTC/TDF arm in regards to efficacy and/or safety [2, 3].

In a large, prospective, treatment-naive trial, subjects with baseline HIV-1 RNA >100 000 c/mL had a lower rate of virologic failure on FTC/TDF compared to 3TC/ABC-containing regimens [3]. Similarly, the BICOMBO study showed that virologically suppressed subjects on a 3TC-containing regimen had a lower rate of virologic failure when switched to FTC/TDF compared to 3TC/ABC-containing regimens [2]. In fact, the 3TC/ABC arm was not noninferior or comparable to the FTC/TDF arm [2]. The total cholesterol (TC), low-density lipoprotein (LDL) cholesterol, and triglycerides (TG) were significantly lower for subjects on FTC/TDF compared to 3TC/ABC [4]. In another study ROCKET 2, virologically suppressed subjects with dyslipidemia on lopinavir/ritonavir (LPV/r) also showed significant declines in TC, LDL, and TG levels 12 weeks following switch to FTC/TDF-compared to 3TC/ABC-containing regimens [5]. Other studies support similar lipid improvement with FTC/TDF [3, 6]. Finally, some but not all studies have shown an association of 3TC/ABC use with an increased relative risk rate of myocardial infarction (MI) [7–13].

US treatment Guidelines list FTC/TDF as a preferred and 3TC/ABC as an alternative NRTI backbone [1, 14]. In light of this, we undertook a prospective, randomized, open-label trial (SWIFT) to evaluate the virologic efficacy and safety potentials and risks of a nucleos(t)ide backbone switch from 3TC/ABC to FTC/TDF in virologically suppressed subjects receiving a ritonavir-boosted protease inhibitor (PI) based regimen.

## METHODS

The SWIFT study was a 48 week prospective, randomized, open–label, multicenter study to evaluate the safety and efficacy of switching FDCs from 3TC/ABC to FTC/TDF in virologically suppressed, HIV-1 infected patients maintained on their boosted PI. Eligible subjects were ≥18 years old, males and nonpregnant females, receiving 3TC/ABC plus a boosted PI with HIV-1 RNA < 200 copies/mL for at least 3 months prior to study entry and < 200 copies/mL at screening by the COBAS TaqMan version 1.0 assay (TaqMan). Subjects had to have an estimated glomerular filtration rate (eGFR) ≥ 50 mL/minutes by the Cockcroft-Gault (CG) method, AST and ALT ≤ 5 times the upper limit of normal, and, if receiving lipid-lowering agents, the drug and dose had to be stable for ≥3 months. Subjects were excluded if they were receiving antiretroviral agents in addition to 3TC/ABC plus a boosted PI, had known historical resistance to any of the study agents including resistance mutations to FTC/TDF (including K65R, M184V/I, or multiple thymidine analogs) or PIs. Subjects were stratified by LPV/r versus other PIs, and by co-morbidities (diabetes mellitus, hyperlipidemia and cardiovascular disease). Antiviral efficacy was assessed by serial measurements of plasma HIV-1 RNA at baseline, and weeks 4, 12, 24, 36, and 48, and at early study discontinuation, if it occurred. Subjects with HIV-1 RNA >200 copies/mL had the test repeated at the investigator's discretion.

The primary objective was to assess non-inferiority of FTC/TDF relative to 3TC/ABC measured by the proportion of subjects who maintained HIV-1 RNA < 200 c/mL through week 48 (intent-to-treat, missing = failure). Secondary objectives included evaluation of safety and tolerability, changes in CD4 cell count, assessment of eGFR using the CG, and the abbreviated modified diet in renal disease (MDRD) methods, and evaluation of change in fasting lipid parameters (TG, TC, LDL, HDL, TC: HDL). In a subset, certain cardiovascular biomarkers (high-sensitivity C-reactive protein [hsCRP], interleukin 6 [IL-6], interleukin 10 [IL-10], tumor necrosis factor α [TNF-α], and fibrinogen) were explored over the 48 weeks. Changes in the risk of coronary heart disease (CHD) outcomes were determined by 10-year Framingham risk scores [15, 16].

## STATISTICAL ANALYSIS

The treated analysis set, used for safety and outcome summaries, includes subjects who were randomized and received at least 1 dose of study drug. The intent to treat (ITT) analysis set, used for efficacy analysis, excludes those with major protocol violations from the treated analysis set.

The primary endpoint was the proportion of subjects with HIV-1 RNA < 200 c/mL through week 48 by time to loss of virologic response (TLOVR) algorithm. TLOVR responders were those who completed the study and maintained HIV-1 RNA < 200 c/mL through week 48 without intervening VF. VF was defined as confirmed on-study HIV-1 RNA ≥ 200 c/mL on 2 successive occasions or the last on-study HIV-1 RNA ≥ 200 c/mL. Subjects were considered failures in the TLOVR analysis if they experienced VF, discontinued study medication before week 48, or changed to a new antiretroviral (ARV) regimen. A 2-sided exact 95% confidence interval (CI) for the difference in treatment group response rate (FTC/TDF minus 3TC/ABC) was constructed using inverted 2 one-sided tests with the standardized statistics. The FTC/TDF group was considered noninferior to the 3TC/ABC group if the lower confidence bound of the responder difference was greater than –12%.

Descriptive statistics summarize secondary efficacy endpoints. Confidence intervals (95%) and tests of significance, all 2-sided were also used for measure of interest of secondary efficacy endpoints. Observed values and changes from baseline in the risk of CHD outcomes for the 10-year Framingham risk score were analyzed. The Framingham risk score was calculated based on using both the fasting TC score and also based on the fasting LDL score approaches. Framingham risk scores were summarized using descriptive statistics and differences between treatment groups and were compared using Wilcoxon rank sum test.

The HIV-1 RNA threshold for VF was amended in the protocol 1 year into the study from 50 c/mL to 200 c/mL based on data regarding discordance between the COBAS Amplicor and the TaqMan HIV-1 test. The data showed an increased rate of samples with >50 c/mL in the TaqMan assay that were < 50 c/mL in the COBAS Amplicor assay [17]. This protocol change was consistent with the ACTG standard of < 200 c/mL [18]. Subjects meeting criteria for VF had genotypic resistance testing performed on their last available plasma sample if HIV-1 RNA >1000 c/mL.

## RESULTS

A total of 312 subjects were randomized from 76 North American centers. One subject randomized to FTC/TDF withdrew consent before receiving study treatment and was excluded from the efficacy and safety analysis. Overall, 311 subjects were randomized and treated (155 started FTC/TDF and 156 continued 3TC/ABC). One subject randomized to FTC/TDF was excluded from the ITT analysis set for a major protocol violation (documented prior resistance to study drug). Demographic and baseline disease characteristics are summarized in Table 1.

**Table 1.** Baseline Demographics and Characteristics

Characteristic	FTC/TDF + PI/r (N = 155)	3TC/ABC + PI/r (N = 156)	Total (N = 311)
Age, median (range), years	46 (22, 66)	46 (22, 75)	46 (22, 75)
Male sex, No. (%)	129 (83)	134 (86)	263 (85)
Race, No. (%)			
White	96 (62)	106 (68)	202 (65)
Black	43 (28)	44 (28)	87 (28)
Asian	4 (3)	3 (2)	7 (2)
Other	12 (8)	3 (2)	15 (5)
Ethnicity			
Hispanic/Latino	38 (25)	36 (23)	74 (24)
Non-Hispanic/Latino	117 (76)	120 (77)	237 (76)
HIV-1 RNA c/mL, No. (%)			
<50	139 (90)	145 (93)	284 (91)
50 to < 200	13 (8)	10 (6)	23 (8)
≥200	3 (2)	1 (1)	4 (1)
Time since first ARV therapy, median (IQR), years	4 (2.5, 6.9)	3.7 (2.5, 6.7)	3.8 (2.5, 6.7)
CD4 cell count, median (IQR), cells/mm^3^	532 (354, 725)	532 (382, 728)	532 (363, 725)
Comorbidities, No. (%)			
Hyperlipidemia	81 (52)	96 (62)	177 (57)
Hypertension	51 (33)	51 (33)	102 (33)
Diabetes	15 (10)	17 (11)	32 (10)
Lipid modifying agent, No. (%)	67 (43)	80 (51)	147 (47)
PI stratification			
Lopinavir/ritonavir	48 (31)	53 (34)	101 (32)
Non-lopinavir/ritonavir	107 (51)	103 (49)	210 (68)
Atazanavir/ritonavir	62 (40)	60 (38)	122 (78)
Fosamprenavir/ritonavir	34 (22)	31 (20)	65 (40)
Darunavir/ritonavir	9 (6)	11 (7)	20 (13)
Other PI	2 (1)	1 (1)	3 (2)
eGFR Cockcroft-Gault, mL/min (IQR)	95 (79–110)	96 (77–113)	…

Abbreviations: ARV, antiretroviral; eGFR, epidermal growth factor receptor; FTC/TDF, emtricitabine/tenofovir disoproxil fumarate; HIV-1, human immunodeficiency virus; IQR, interquartile range; PI, protease inhibitor; 3TC/ABC, lamivudine/abacavir.

### Efficacy Results

**Figure 1.**
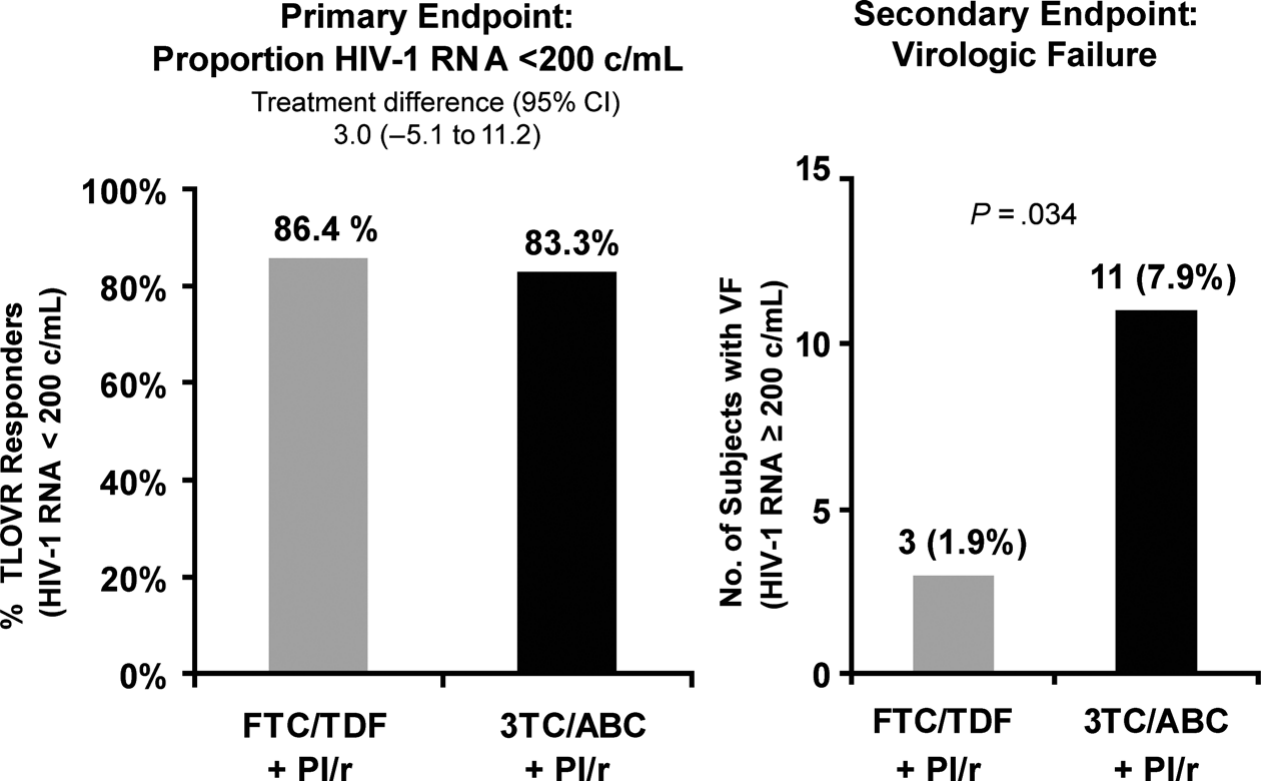
Virologic response and virologic failure by Kaplan-Meier through week 48.

Abbreviations: CI, confidence interval; FTC/TDF,emtricitabine/tenofovir disoproxil fumarate; HIV-1, human immunodeficiency virus type 1; PI, protease inhibitor; 3TC/ABC, lamivudine/abacavir; TLOVR, time to loss of virologic response.

At week 48, TLOVR responses were 133 of 155 (86.4%) for the FTC/TDF arm compared to 130 of 156 (83.3%) with continued 3TC/ABC, representing a treatment difference of 3.0% (95% CI, −5.1% to 11.2%), establishing noninferiorty. Additionally, fewer people had virologic failure in the FTC/TDF arm vs 3TC/ABC, 3/155 (1.9%) vs 11/156 (7.1%); *P* = .034 through week 48 (Figure 1). All 3 subjects who experienced virologic failure in the FTC/TDF arm had low-level viremia (range, 209–452 copies/mL); low adherence was not reported in these subjects with low-level viremia. Two were receiving atazanavir/ritonavir and 1 boosted fosamprenavir. Of the 11 subjects with virologic failure in the 3TC/ABC arm, 3 discontinued study drug early, and 8 subjects experienced viremia (range, 272–6430 copies/mL) at the week 48 visit. Of these 11 subjects, 5 were receiving atazanavir/ritonavir, 4 lopinavir/ritonavir, 1 fosamprenavir/ritonavir, and 1 darunavir/ritonavir. No specific boosted PI regimen was associated with virologic failure.

Four virologic failure subjects had HIV-1 RNA values above 1000 copies/mL and had genotypic and phenotypic analyses: 1 subject in the FTC/TDF arm and 3 subjects in the 3TC/ABC arm. No genotypic resistance to study drugs was observed in any subject in either arm through week 48. Note, of the 4 subjects who were suppressed at screening but above the HIV-1 RNA value of 200 copies/mL at baseline, 2 were virologic successes due to post-baseline ongoing virologic suppression, 1 was a virologic failure due to detectable but low-level viremia at week 48 while on FTC/TDF, and 1 subject was excluded from the ITT analysis set due to a major protocol violation.

Changes in CD4 count at week 48 were similar between treatment arms with median (IQR) changes of 8 (−49, 80) and 39 (−41, 125) cells/mm^3^ for the FTC/TDF and the 3TC/ABC arms, respectively (*P* = .10).

Subjects who switched to FTC/TDF from 3TC/ABC showed reductions from baseline at week 48 in fasting TC (median change of −21 mg/dL vs −3 mg/dL with 3TC/ABC, *P* < .001), and LDL (−7 mg/dL vs 2 mg/dL with 3TC/ABC; *P* = .007). There were no differences in lipid lowering agent modification between arms during the study. No differences in HDL (*P* = .26), TG (*P* = .074) or HDL/TC ratio (*P* = .17) were observed (Supplement 1).

**Figure 2.**
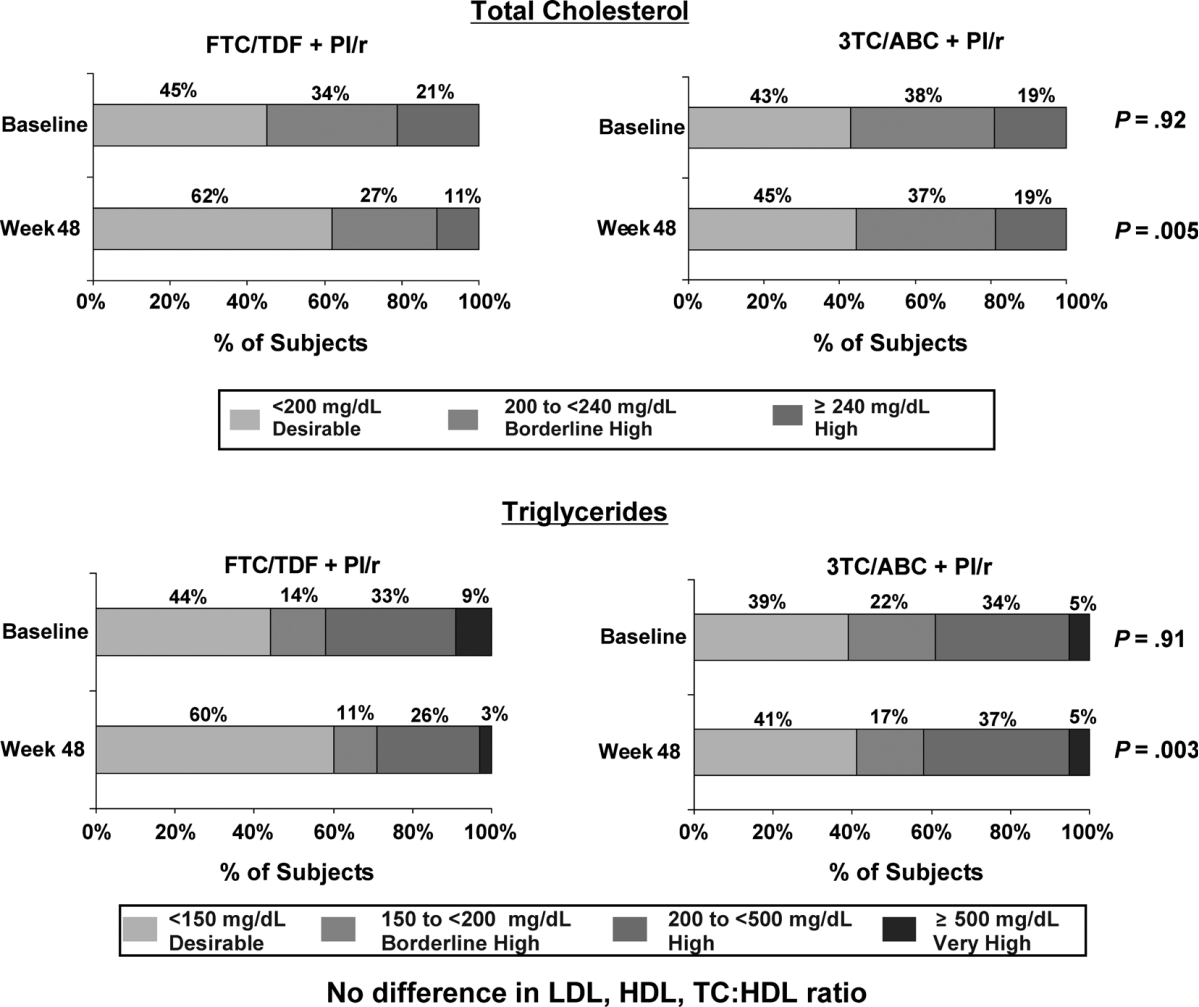
Fasting total cholesterol and triglycerides by National Cholesterol Education Program classification.

Abbreviations: FTC/TDF, emtricitabine/tenofovir disoproxil fumarate; PI, protease inhibitor; 3TC/ABC, lamivudine/abacavir.

At baseline, there was no difference in the distribution across National Cholesterol Education Program (NCEP) categories between the 2 treatment groups; NCEP sets cholesterol guidelines in the United States [16]. At week 48, a higher percentage of subjects who switched to FTC/TDF were in the desirable NCEP categories for TC and TG compared to those who remained on 3TC/ABC (TC: 62% vs 45% < 200 mg/dL, *P* = .005; TG: 60% vs 41% < 150 mg/dL, *P* = .003) (Figure 2).

**Figure 3.**
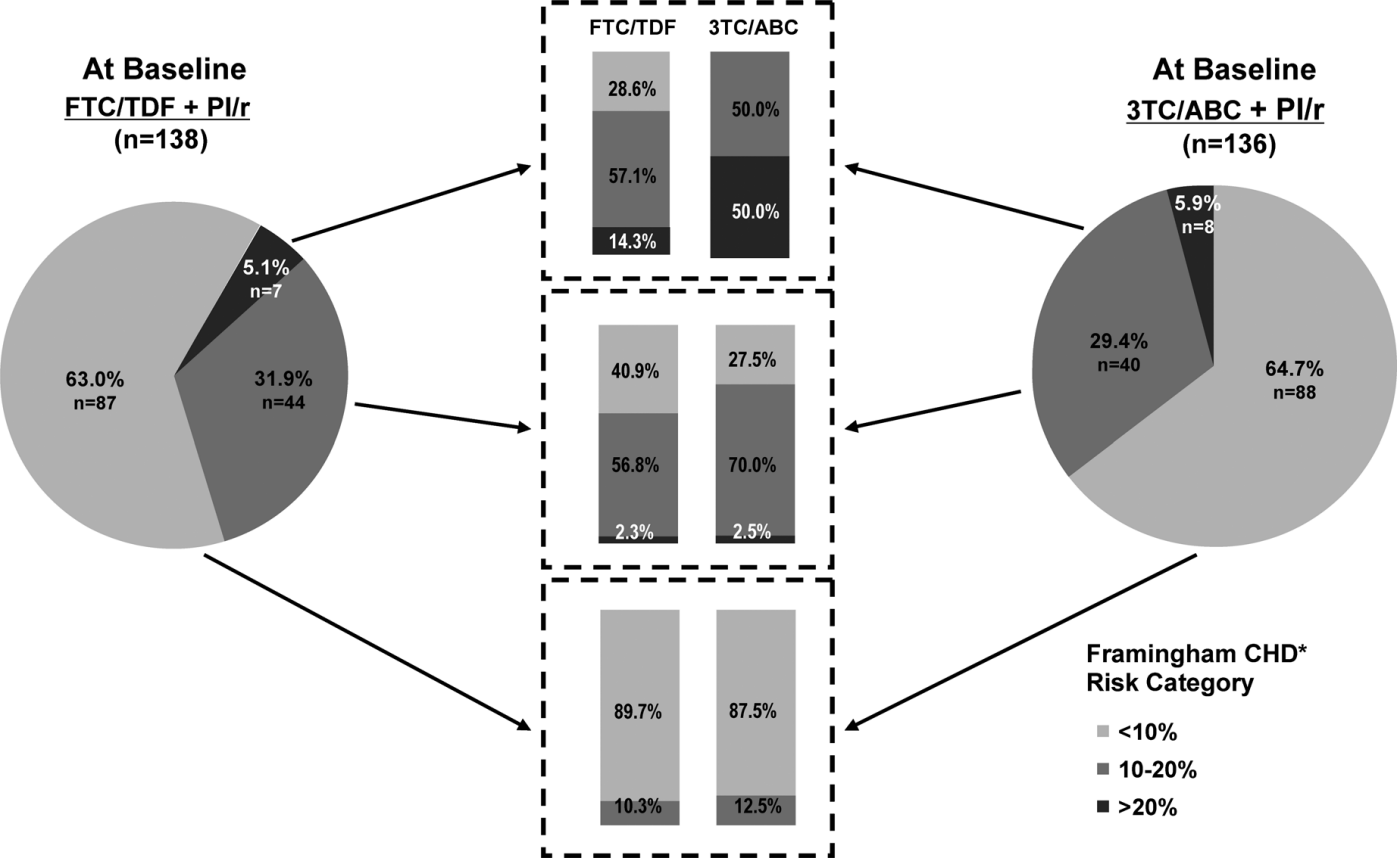
Categorical shifts by Framingham 10-year risk scores from baseline to week 48.

Abbreviations: CHD, coronary heart disease; FTC/TDF, emtricitabine/tenofovir disoproxil fumarate; PI, protease inhibitor; 3TC/ABC, lamivudine/abacavir.

Switching to FTC/TDF resulted in improvements in the predicted risk for CHD outcomes as measured by Framingham Risk Scores. Mean (SD) change from baseline in risk by the TC formula was −1.0 (4.32) for the FTC/TDF arm at week 12 (*P* = .008); this reduction was also maintained through week 48 with a mean (SD) change from baseline of −1.2 (4.39) and *P* = .006. When the LDL formula was used, mean (SD) change from baseline in Framingham risk was −0.9 (3.07) for the FTC/TDF arm at week 12 (*P* < .001) and was −0.5 (3.93) at week 48 (*P* = .21). The mean change for all calculated Framingham Scores in the 3TC/ABC group fluctuated about the baseline level with no statistically significant changes from baseline observed. The difference between groups for the predicted risk of CHD (regardless of method of calculation) only achieved statistical significance at week 24 (*P* < .05). The FTC/TDF group further demonstrated a shift from higher risk Framingham categories to lower risk categories (Figure 3).

### Adverse Events

The safety and tolerability for both treatment arms in SWIFT were consistent with the known safety profiles of FTC/TDF and 3TC/ABC (Table 2). Similar percentages of subjects in each arm reported any serious adverse event (SAE), any adverse event (AE), or any Grade 3 or 4 treatment-emergent AE. Three subjects died during the study: 1 subject in the FTC/TDF group (suicide) and 2 subjects in the 3TC/ABC group (homicide, lymphoma). None of the deaths or SAEs was considered by the investigator to be related to study (Table 3). There was one pregnancy in the 3TC/ABC arm with a spontaneous abortion, which was considered unrelated to the study drug.

**Table 2.** Summary of Adverse Events (Treated Analysis Set)

Adverse Event Category, No. (%)^a^	FTC/TDF + PI/r (N = 155)	3TC/ABC + PI/r (N = 156)	Total (N = 311)
Adverse event	112 (72.3%)	120 (76.9%)	232 (74.6%)
Grade 3 or 4 adverse event	13 (8.4%)	16 (10.3%)	29 (9.3%)
Adverse event related to study drug	16 (10.3%)	6 (3.8%)	22 (7.1%)
Grade 3 or 4 adverse event related to study drug	1 (0.6%)	0	1 (0.3%)
Serious adverse event	12 (7.7%)	11 (7.1%)	23 (7.4%)
Serious adverse event related to study drug	0	0	0
Adverse event leading to study drug discontinuation	7 (4.5%)	3 (1.9%)	10 (3.2%)
Death during study	1 (0.6%)	2 (1.3%)	3 (1.0%)

Abbreviations: FTC/TDF,emtricitabine/tenofovir disoproxil fumarate; PI,protease inhibitor; 3TC/ABC,lamivudine/abacavir.

**Table 3.** Disposition of Subjects

Subject Disposition^a^	FTC/TDF + PI/r	3TC/ABC + PI/r	Total
Subjects randomized	156	156	312
Subjects randomized but not treated	1	0	1
Subjects treated	155	156	311
Completed 48 weeks of study^b^	138 (89.0)	139 (89.1)	277 (89.1)
Discontinued study drug prematurely	17 (11.0)	17 (10.9)	34 (10.9)
Primary reason for premature discontinuation of study			
Adverse event	7 (4.5)	3 (1.9)	10 (3.2)
Death	0	0	0
Pregnancy	0	1 (0.6)	1 (0.3)
Lack of efficacy	0	1 (0.6)	1 (0.3)
Investigator's discretion	0	3 (1.9)	3 (1.0)
Withdrew consent	5 (3.2)	4 (2.6)	9 (2.9)
Lost to follow-up	4 (2.6)	5 (3.2)	9 (2.9)
Subject noncompliance	0	0	0
Protocol violation	1 (0.6)	0	1 (0.3)
Study discontinued by sponsor	0	0	0

Abbreviations: FTC/TDF,emtricitabine/tenofovir disoproxil fumarate; PI,protease inhibitor; 3TC/ABC,lamivudine/abacavir.

^a^ All percentages are based on the No. of subjects in the treated analysis set.

^b^ Subjects completed 48 weeks of the study if the subject completed the protocol-planned duration of the study based on the study completion form.

The percentage of subjects who discontinued study drug due to an AE was higher in the FTC/TDF group [4.5% (*n* = 7/155)], compared to the 3TC/ABC [1.9% (*n* = 3/156)]. Rash, which was reported in 1.3% (2 subjects) in the FTC/TDF arm, was the only AE reported in more than 1 subject that resulted in study drug discontinuation. A higher percentage of treatment-emergent AEs considered related to study drug by the investigator were reported in the FTC/TDF than in the 3TC/ABC group, 10.3% (*n* = 16) vs 3.8% (*n* = 6). Adverse events considered related to the study drug in more than 1 subject included nausea, headache, and dizziness (1.9%, 3 subjects each); diarrhea, flatulence, malaise, and rash (1.3%, 2 subjects each) in the FTC/TDF group; and diarrhea (1.3%, 2 subjects) in the 3TC/ABC group.

There were no differences observed in renal adverse events between arms (FTC/TDF 4.5% [*n* = 7]; 3TC/ABC 5.1% [*n* = 8]). Three subjects in the FTC/TDF arm had renal AEs reported as related to study drug by the investigator: renal impairment (baseline serum creatinine [SCr] of 1.0 mg/dL which subsequently increased to 1.3 mg/dL then decreased to 1.1 mg/dL), abnormal urine odor, and increased SCr with decreased eGFR (grade 1 decrease at discontinuation).

Modest decreases from baseline through week 48 in creatinine clearance by the CG method (GFR_CG_) using ideal body weight occurred within both treatment arms, FTC/TDF (GFR_CG_−8.3 mL/minutes, *P* < .001) and 3TC/ABC (GFR_CG_ −4.5 mL/minutes, *P* = .002). When compared across arms, a statistically significant difference was observed between the groups (*P* = .012). MDRD GFR estimates gave similar results (Supplement 2).

Treatment emergent laboratory abnormalities were comparable between the groups. Most laboratory abnormalities were grade 1 or 2, and most common was elevated bilirubin, primarily in subjects on ATV + RTV. There was no grade 2 or higher changes in SCr throughout the study. Grade 1 SCr laboratory changes occurred in 3.2% of subjects on FTC/TDF and 1.9% on 3TC/ABC. No clinically relevant changes in serum phosphorus and in hypophosphatemia were observed. There was no difference in development of proteinuria between the 2 arms when analyzed by change in grade from baseline (Table 4). No patients had confirmed normoglycemic glucosuria in either arm.

**Table 4.** Change From Baseline in Urine Protein by Grade

Urine Protein Change in Grade^a^	−2	−1	0	+1	+2	+3
FTC/TDF (n = 148)	2	11	107	25	3	…
3TC/ABC (n = 151)	1	15	114	21	0	…
Total (N = 299)	3	26	221	46	3	…

Cochran-Mantel-Haenssel statistics (based on table scores).

Abbreviations: FTC/TDF,emtricitabine/tenofovir disoproxil fumarate; 3TC/ABC,lamivudine/abacavir.

^a^ Nonzero correlation value 1.5674, *P* = .2106; row mean scores diff 1.5674, *P* = .2106.

Given previous reports of increased risks for cardiovascular events, including myocardial infarction, associated with ARV regimens containing ABC, we explored changes in commonly used surrogate cardiovascular biomarkers in a subset of 159 of 312 (51%) patients, 81 randomized to FTC/TDF and 78 to 3TC/ABC. No differences at week 48 compared to baseline were observed between treatment arms for hsCRP, IL-10, IL-6, and TNF-α (Table 5), although there was a trend for differences in fibrinogen (median change, FTC/TDF −10 mg/dL, 3TC/ABC −1 mg/dL, *P* = .062) at week 48.

**Table 5.** Cardiovascular Biomarkers Change From Baseline at Week 48^a^

Cardiovascular Biomarkers, Median (Q1, Q3)	FTC/TDF + PI/r	3TC/ABC + PI/r
C-reactive protein (mg/dL)	N = 69 −0.013 (−0.123, 0.054)	N = 57 0.006 (−0.078, 0.113)
	*P* = .19	
Fibrinogen (mg/dL)	N = 64 −10 (−50, 31)	N = 56 −1 (−19, 54)
	*P* = .062	
IL-10-INF (pg/mL)	N = 68 0.0 (0.0, 0.0)	N = 56 0.0 (−0.4, 0.0)
	*P* = .22	
IL-6-INF (pg/mL)	N = 68 0.0 (−0.4, 0.2)	N = 56 0.0 (−1.2, 0.2)
	*P* = .58	
TNF-α-INF (pg/mL)	N = 68 0.0 (0.0, 0.0)	N = 56 0.0 (0.0, 0.0)
	*P* = .69	

*P*-values for comparison between treatment groups are from Wilcoxon rank-sum test.

Abbreviations: 3TC/ABC, lamivudine/abacavir; FTC/TDF, emtricitabine/tenofovir disoproxil fumarate; IL, interleukin; INF, interferon; PI, protease inhibitor; TNF, tumor necrosis factor.

^a^ Missing = excluded analysis.

## DISCUSSION

In this large, prospective, randomized trial, the first study specifically designed to evaluate the efficacy and safety of switching from 3TC/ABC to FTC/TDF in HIV-1-infected subjects suppressed on a PI + RTV containing regimen, we have demonstrated that FTC/TDF is noninferior to remaining on 3TC/ABC in maintaining treatment response by TLOVR with fewer VFs, as well as a lower risk of emergent resistance through 48 weeks. Efficacy and virologic failure rates in subjects on FTC/TDF compared to 3TC/ABC arm were comparable to results seen with this FDC seen in the BICOMBO and ASSERT trials [2, 19].

We did observe slightly higher rates of discontinuation due to AEs and mild AEs considered study drug-related in the FTC/TDF vs 3TC/ABC arms. This finding is not unexpected as previous studies demonstrate an increase in certain adverse events when stable subjects are switched to a new therapy. Modest declines in eGFR occurred in both arms with the degree of decline significantly greater in FTC/TDF-treated subjects; however, values remained in the normal range. Long-term studies have shown that the use of TDF may be associated with initial declines in GFR within the first few months of starting TDF, which then stabilize [20–22]. Importantly, there were no differences between arms in emergent proteinuria and or normoglycemic glycosuria (Table 4).

Comorbidities (FTC/TDF vs 3TC/ABC) were common in our study population, including diabetes (10% vs11%), hyperlipidemia (52% vs 62%), and hypertension (33% vs 33%). Additionally, 3.2% in both arms had a history of MI. As with previous studies, we demonstrated lipid benefits when switching to FTC/TDF [3–6]. Significant declines in LDL and TC were observed by week 12 in the FTC/TDF arm and significant reductions in TC, LDL, and TG were seen at week 48. By NCEP category criteria [16], higher percentages showed improvements in TC and LDL, as well as improvement (shift from a higher risk to a lower risk category) in the predicted risk for CHD outcomes with FTC/TDF as seen in other comparative studies [2, 5]. In an ad hoc analysis, we found that the predicted Framingham 10-yr Risk Score was more favorable when switching FTC/TDF; particularly, for those with comorbidities, whites, and regimens with a PI other than LPV/r. Such an improvement in Framingham scores is perhaps one of the most novel benefits of switching from an 3TC/ABC to FTC/TDF-containing regimen. It is however, worthwhile to note that the small number of subjects on LPV/r and other confounding factors at baseline may make this a weaker correlation and may limit these results from being generalized. Finally, the changes from baseline in commonly used surrogate cardiovascular biomarkers (hsCRP, IL-10, IL-6, TNF-α, and fibrinogen) between the FTC/TDF and 3TC/ABC arm in a subset of 159 subjects were not significant except a trend toward significance with fibrinogen (*P* = .062) (Table 5), perhaps with a larger sample size it may have achieved significance.

The SWIFT study showed that high rates of virologic suppression were well maintained through 48 weeks with fewer virologic failures in subjects who switched to FTC/TDF, and also this regimen is well tolerated. Decreases in creatinine clearance did occur with both treatments and were greater in the FTC/TDF arm. In the FTC/TDF arm, improvements in certain lipid parameters and in other measures including in NCEP categories and Framingham predicted risk for CHD outcomes were noted [15, 16]. In summary, switching patients on a boosted PI regimen to FTC/TDF from 3TC/ABC is associated with important metabolic benefits without loss of virologic control.

## Supplementary Data

Supplementary materials are available at *Clinical Infectious Diseases* online (http://cid.oxfordjournals.org/). Supplementary materials consist of data provided by the author that are published to benefit the reader. The posted materials are not copyedited. The contents of all supplementary data are the sole responsibility of the authors. Questions or messages regarding errors should be addressed to the author.

Supplementary Data
