# Supplementary material for: SWIFT: Prospective 48-Week Study to Evaluate Efficacy and Safety of Switching to Emtricitabine/Tenofovir From Lamivudine/Abacavir in Virologically Suppressed HIV-1 Infected Patients on a Boosted Protease Inhibitor Containing Antiretroviral Regimen
Source: Clin Infect Dis. 2013 Jan 29;56(11):1637–45. doi: 10.1093/cid/cis1203 (PMC3641864; doi:10.1093/cid/cis1203)
Supplement: Supplementary Data [file supp_56_11_1637__index.html]

SWIFT: Prospective 48 Week Study to Evaluate Efficacy and Safety of Switching to Emtricitibine/Tenofovir from Lamivudine/Abacavir in Virologically Suppressed HIV-1 Infected Patients on a Boosted Protease Inhibitor Containing Antiretroviral Regimen — SWIFT: Prospective 48-Week Study to Evaluate Efficacy and Safety of Switching to Emtricitibine/Tenofovir From Lamivudine/Abacavir in Virologically Suppressed HIV-1 Infected Patients on a Boosted Protease Inhibitor Containing Antiretroviral Regimen — SWIFT: Prospective 48-Week Study to Evaluate Efficacy and Safety of Switching to Emtricitabine/Tenofovir From Lamivudine/Abacavir in Virologically Suppressed HIV-1 Infected Patients on a Boosted Protease Inhibitor Containing Antiretroviral Regimen — Supplementary Data 

# SWIFT: Prospective 48-Week Study to Evaluate Efficacy and Safety of Switching to Emtricitabine/Tenofovir From Lamivudine/Abacavir in Virologically Suppressed HIV-1 Infected Patients on a Boosted Protease Inhibitor Containing Antiretroviral Regimen

## Supplementary Data

Supplementary Data

**Files in this Data Supplement:**

- Supplementary Figure 1 - eps file
- Supplementary Figure 2 - eps file
